# Abnormal Topological Network in Parkinson’s Disease With Impulse Control Disorders: A Resting-State Functional Magnetic Resonance Imaging Study

**DOI:** 10.3389/fnins.2021.651710

**Published:** 2021-08-23

**Authors:** Xiaopeng Zhu, Langsha Liu, Yan Xiao, Fan Li, Yongkai Huang, Deqing Han, Chun Yang, Sian Pan

**Affiliations:** ^1^Department of Neurosurgery, Zhuzhou Central Hospital, Zhuzhou, China; ^2^Department of Cardiac Surgery, Xiangya Hospital, Central South University, Changsha, China; ^3^Department of Day Surgery Center, Zhuzhou Central Hospital, Zhuzhou, China; ^4^Department of Rehabilitation Medicine, Zhuzhou Central Hospital, Zhuzhou, China

**Keywords:** Parkinson’s disease, impulse control disorders, fMRI, topological network, graph theory

## Abstract

In recent years, neuroimaging evidence shows that the brains of Parkinson disease (PD) with impulse control disorders (ICDs) patients have functional disconnection changes. However, so far, it is still unclear whether the topological organization is damaged in PD patients with ICD. In this study, we aimed to explore the functional brain network in 18 patients with PD with ICDs (PD-ICD) and 18 patients with PD without ICDs (PD-nICD) by using functional magnetic resonance imaging and graph theory approach. We found that the PD-ICD patients had increased clustering coefficient and characteristic path length, while decreased small-world index compared with PD-nICD patients. Furthermore, we explored the hypothesis whether the abnormality of the small-world network parameters of PD-ICD patients is accompanied by the change of nodal centrality. As we hypothesized, the nodal centralities of the default mode network, control network, and dorsal attention network were found to be significantly damaged in the PD-ICD group compared with the PD-nICD group. Our study provides more evidence for PD-ICD patients’ brain network abnormalities from the perspective of information exchange, which may be the underlying pathophysiological basis of brain abnormalities in PD-ICD patients.

## Introduction

Parkinson disease (PD) is a common neurodegenerative disease in middle-aged and elderly people. Its main clinical manifestations are motor symptoms such as resting tremor, bradykinesia, muscle rigidity, and abnormal posture, as well as non-motor symptoms such as impulse control disorders (ICDs). ICD refers to a mental disorder in which patients are driven by a strong desire to adopt improper behaviors to obtain self-satisfaction. Fifteen percent of PD patients have one or more clinical symptoms of ICD (Vriend, 2018), which mainly include pathological gambling, compulsive eating, hypersexuality, compulsive shopping, and so on (Vargas and Cardoso, 2018). Once PD patients suffer from ICD, clinical management and intervention will become more difficult. Therefore, in the early stage of PD with ICDs (PD-ICD), it is very important to understand the corresponding specific changes of PD-ICD.

Many previous neuroimaging studies, involving brain metabolism by using single-photon emission tomography (SPECT) (Cilia et al., 2011) and positron emission tomography (Thiel et al., 2003) and morphometric and functional imaging by using magnetic resonance imaging (MRI) (Frosini et al., 2010; Carriere et al., 2015; Tessitore et al., 2016), have made a lot of contributions in exploring the abnormal changes related to PD-ICD. They have consistently demonstrated dysfunction in both cortical and subcortical areas, which are important in the reward system, such as the striatum, anterior cingulate cortex (ACC), and insula. For example, in the SPECT study, PD patients with pathological gambling were found to be associated with ACC–striatal disconnection, which may indicate a specific abnormality of behavior control and explain why PD gamblers used to persist in gambling despite the self-destructive consequences (Cilia et al., 2011). A recent review of functional studies revealed decreased activity in ACC and increased activity in the ventral striatum and orbitofrontal cortex (Santangelo et al., 2019). Evidence from one of the resting-state functional MRI (rs-fMRI) studies showed that PD with ICD symptoms was associated with the functional disconnection between the left anterior putamen (an associative striatal area) and the left inferior temporal gyrus and the left ACC (limbic cortical regions) (Carriere et al., 2015). In addition, PD-ICD patients have been shown through several diffusion tensor imaging tractography to have widespread white matter tract damage (Yoo et al., 2015; Canu et al., 2017; Zadeh et al., 2018), which further confirm the impaired network connection in PD patients with ICD.

In recent years, as a new network analysis method, graph theory analysis has been widely used in the research of many neurological and psychiatric diseases including PD. This method models the brain regions and the connections between regions as nodes and edges, such that the brain is modeled as a topological network composed of many points and edges, which can be studied for network parameters and network efficiency. However, so far, it is still unclear whether the topographic organization is damaged in PD patients with ICD. Given the disconnected brain in PD with ICD, as well as the disruption of topological organization in PD, it is plausible that the whole-brain topological network of PD-MCI may also be damaged in a diseased state. Therefore, in the current study, we aimed to explore whether the presence of ICD in PD patients may determine abnormalities in the topological network by using rs-fMRI and graph theory methods.

## Materials and Methods

### Study Population

All MRI and experimental data used in this study were obtained from the Parkinson’s Progression Markers Initiative,^1^ which is a large-scale, comprehensive observational, multicenter project of PD progression biomarkers (Marek et al., 2011). A total of 52 participants were analyzed, including 18 participants in the PD-ICD group, 18 in the PD without ICDs (PD-nICD) group, and 16 age- and sex-matched health control (HC) group (Table 1). All PD patients were diagnosed according to the criteria of the United Kingdom Brain Bank (Hughes et al., 1992). The study was approved by the Institutional Review Boards/Independent Ethics Committees. Written informed consent was obtained from all subjects. For more details on the study, see http://www.ppmi-info.org/wp-content/uploads/2013/02/PPMI-Protocol-AM5-Final-27Nov2012v6-2.pdf.

**TABLE 1:** Demographic and clinical features of HC, PD-nICD, and PD-ICD.

	HC (*n* = 16)	PD-nICD (*n* = 18)	PD-ICD (*n* = 18)	*p*-Value
Age (years)	64.75 ± 9.28	59.69 ± 11.64	62.13 ± 12.53	0.44
Gender (male:female)	13:3	12:6	12:6	0.56
Disease duration (years)	–	1.90 ± 0.81	2.62 ± 1.33	0.06
UPDRS-III	–	19.67 ± 8.22	22.39 ± 10.72	0.40
Hoehn and Yahr stage	–	1.78 ± 0.43	1.83 ± 0.51	0.73
Montreal cognitive assessment	27.75 ± 1.25	27.44 ± 2.25	25.67 ± 4.39	0.10
Education (years)	16.88 ± 2.66	15.56 ± 2.91	15.78 ± 2.96	0.37
Depression (Geriatric Depression Scale 15)	2.19 ± 2.95	2.06 ± 1.92	3.11 ± 2.61	0.40
ICD (Questionnaire for Impulsive–Compulsive Disorders in Parkinson Disease–Current Short)	0 ± 0	0 ± 0	1.22 ± 0.55	<0.00001

### MRI Parameters

The imaging data were acquired on the Siemens 3-T MRI scanner. T1-weighted gradient-echo 3D MPRAGE sequence was used [repetition time (TR) = 2,300 ms, time to echo (TE) = 2.98 ms, fractional anisotropy (FA) = 9°, 1-mm^3^ isotropic voxel] to obtain high-resolution structural images. rs-fMRI scan is obtained by echo plane sequence (TR = 2,400 ms, TE = 25 ms, FA = 80, voxel size = 3.3 mm^3^, total 210 volumes, 40 axial slices). It is recommended that the subjects open their eyes and relax quietly to perform a functional scan of the resting state and try not to fall asleep.

### Data Preprocessing and Statistical Analysis

Results included in this manuscript come from preprocessing performed using fMRIPrep 1.4.1 (Esteban et al., 2019), which is based on Nipype 1.2.0 (Gorgolewski et al., 2011). More method details are shown in the Supplementary Material.

## Results

### The Small-World Topology in PD-ICD, PD-nICD, and HC Patients

**FIGURE 1:**
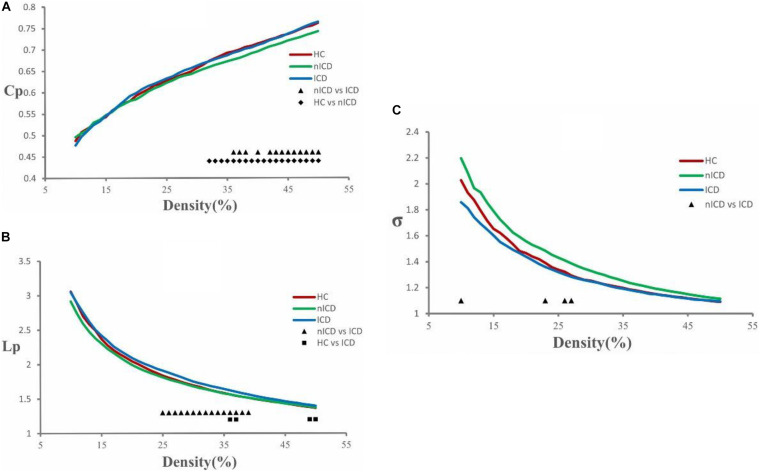
Cluster coefficient (Cp), characteristic path length (Lp), and small-world index (σ) in PD-nICD, PD-ICD, and HC. **(A)** The Cp from three groups. Black triangle means significant differences between PD-nICD and PD-ICD (36–38, 40, 42–50%, *p* < 0.05). Black diamond means significant differences between HC and PD-nICD (density thresholds: 32–50%, *p* < 0.05). **(B)** The Lp from three groups. Black triangle means significant differences between PD-nICD and PD-ICD (density thresholds: 25–39%, *p* < 0.05). Black square means significant differences between HC and PD-ICD (density thresholds: 36–37, 49–50% *p* < 0.05). **(C)** The σ from three groups. Black triangle means significant differences between PD-nICD and PD-ICD (density thresholds: 10, 23, 26–27%, *p* < 0.05).

The topological brain networks at all three groups had the characteristics of “small-world” networks. For example, over an entire range of density thresholds, the small-world indexes of these three groups were larger than one (σ > 1) (Figure 1C).

### PD-ICD Group Versus PD-nICD Group

Compared to the PD-nICD group, the PD-ICD group showed significantly increased clustering coefficient Cp (density thresholds: 36–38, 40, 42–50%, *p* < 0.05, two-tailed) (Figure 1A), characteristic path length Lp (density thresholds: 25–39%, *p* < 0.05, two-tailed) (Figure 1B), and significantly decreased small-world index σ (density thresholds: 10, 23, 26–27%, *p* < 0.05, two-tailed) (Figure 1C). Furthermore, we explored the hypothesis whether the abnormality of the small-world network parameters of PD-ICD patients is accompanied by the change of nodal centrality. Compared with the PD-nICD group, the brain regions with significantly increased node centrality in PD-ICD patients are located in default mode network (DMN), control network (CN), and dorsal attention network (DAN), and the significantly reduced regions are located in DAN (Table 2).

**TABLE 2:** Nodal centrality differences between PD-ICD and PD-nICD.

	ROI label	ROI name	ROI network	*p*-Value
PD-ICD larger than PD-nICD	36	7Networks_LH_Cont_pCun_1	CN	0.001
	49	7Networks_LH_Default_pCunPCC_1	DMN	0.004
	67	7Networks_RH_DorsAttn_Post_1	DAN	0.037
PD-ICD smaller than PD-nICD	71	7Networks_RH_DorsAttn_Post_5	DAN	0.049

### PD-nICD Group Versus HC Group

Compared to the HC group, the PD-nICD group showed a significantly decreased clustering coefficient Cp (density thresholds: 32–50%, *p* < 0.05, two-tailed) (Figure 1A).

### PD-ICD Group Versus HC Group

Compared to the HC group, the PD-ICD group showed significantly increased characteristic path length Lp (density thresholds: 36–37, 49–50% *p* < 0.05, two-tailed) (Figure 1B).

## Discussion

As far as we know, this is the first time to explore the changes of the brains from the perspective of topological networks in PD patients with ICD by using fMRI and graph theory analysis. We found that the PD-ICD patients had increased clustering coefficient and characteristic path length, while decreased small-world index compared with PD-nICD patients. Furthermore, we explored the hypothesis whether the abnormality of the small-world network parameters of PD-ICD patients is accompanied by the change of nodal centrality. As we hypothesized, the nodal centralities of DMN, CN, and DAN were found to be significantly damaged in the PD-ICD group compared with the PD-nICD group. As the pathogenesis of PD-ICD is not yet fully understood, we will further discuss our findings from the perspective of the topological network and the underlying pathophysiological basis that may arise.

Our results showed that whether it is NC group’s, PD-nICD group’s, or PD-ICD group’s brain functional network, they are all in line with the characteristics of small-world networks. This is similar to the studies of topological networks in other brain diseases, such as Alzheimer disease, schizophrenia, and so on (Liu et al., 2008; Zhao et al., 2012; Seo et al., 2013). Especially in the recent related research of topological networks in PD patients, the small-world network characteristics of the PD brain are also consistently presented (Luo et al., 2014; Berman et al., 2016; Chen et al., 2020). A small-world network is a relatively high-efficiency network model, with a high clustering coefficient and low characteristic shortest path length (Bullmore and Sporns, 2009). The brains of PD-ICD patients also have the characteristics of a small-world network, indicating that even in a disease state, the brain network is still a relatively efficient network model, which may be necessary for their daily activities, such as recalling, thinking, or decision making.

Although the brain network of PD-ICD patients presented a small-world characteristic, the network parameters were significantly different from those of the PD-nICD group. In our study, the clustering coefficient and characteristic shortest path length of the functional network in PD-ICD were significantly higher than in PD-nICD. In a topological network, the clustering coefficient reflects the efficiency of information transfer between local areas, and the higher the clustering coefficient, the more efficient the integration of information in the local area. The characteristic shortest path length reflects the overall information transmission, and the shorter the characteristic shortest path, the higher the efficiency of information transmission between long-distance areas (Bullmore and Sporns, 2009). Therefore, the increase in the clustering coefficient of the functional network of PD-ICD patients may indicate that the communication between the local brain regions related to impulsive behaviors is enhanced, leading to the occurrence of impulsive behaviors. The increase in the patient’s characteristic path length may indicate that the information exchange in the remote brain regions that inhibit impulsive behavior has also become slower, making it more difficult for patients to control and stop these impulsive behaviors.

We also explored the hypothesis whether the abnormality of clustering coefficient and characteristic shortest path length in PD-ICD patients is accompanied by the change of nodal centrality. Our results show that, compared with PD-nICD, the brain regions with significantly increased node centrality in PD-ICD patients are located in DMN, CN, and DAN, and the significantly reduced regions are located in DAN.

Default mode network, CN, and DAN are three networks that play an important role in cognition, behavior, and attention, which are inseparable from the brain’s information exchange and processing (Petrides, 2005; Fox et al., 2006; Koechlin and Summerfield, 2007; Buckner et al., 2008; Spreng et al., 2009). The DMN is considered to be related to ruminations, mind-wandering, and cognitive processing (Buckner et al., 2008; Spreng et al., 2009). The CEN is involved in the process of external stimuli, decision-making, and executive behaviors (Petrides, 2005; Koechlin and Summerfield, 2007). DAN is one of the sensory orientation systems in the human brain. It involves voluntary top-down orientation and indicates when, where, or in what direction the subject should perform behavioral activities (Fox et al., 2006). When it comes to PD with ICD symptoms, there are a few previous articles about these networks. As far as we know, there are currently only two related articles. One of them found that PD-ICD was related to increased connectivity within the salience network (SN) and DMN, as well as with a decreased connectivity within CN (Tessitore et al., 2017b). The other found decreased connectivity in DMN and CN, and increased connectivity in SN in PD-ICD when compared with PD-nICD patients (Tessitore et al., 2017a). Similar to previous studies, the current article also found that the DMN and CN network in the brain of PD-ICD patients are significantly abnormal compared with PD-nICD. The difference is that our research did not find significant changes in the SN network but found that the DAN network was damaged. This may be because our research method used a large-scale topological network, the information flow related to the centrality of the node needs to calculate the flow of information in the whole brain, rather than the functional connection between several brain regions. However, it is worth noting that our research also found DMN and CN in the brains of PD-ICD patients from the perspective of the topological network. This may indicate that DMN and CN network abnormalities are related to the pathogenesis and development of ICD symptoms in PD, and whether there are related abnormalities in DAN network still needs more research to confirm.

This study still has some limitations. The most important aspect is that the sample size is relatively small. Although our research found significant topological network differences between PD-ICD and PD-nICD, the small sample size prevented us from obtaining multiple comparisons corrected results. Second, we control the influence of confusion factors (such as age, gender, and so on) between groups on the topological network, but we cannot control the influence of these factors within the group. Third, the neural network mechanisms of different subtypes of ICD may be different. This study also did not distinguish the impact of ICD subtypes on topological networks. We hope that sufficient samples can be obtained in future studies for further research.

## Conclusion

In summary, by using the topological network analysis method, we found that the clustering coefficient and characteristic path length of the brain function network of PD-ICD patients increased, accompanied by damage to the DMN, CN, and DAN network nodes. This may be the underlying pathophysiological basis of brain abnormalities in PD-ICD patients. At the same time, the current study also provides more evidence for PD-ICD patients’ brain network abnormalities from the perspective of information exchange.

## Data Availability Statement

The original contributions presented in the study are included in the article/Supplementary Material, further inquiries can be directed to the corresponding author/s.

## Ethics Statement

The studies involving human participants were reviewed and approved by the Zhuzhou Central Hospital. The patients/participants provided their written informed consent to participate in this study.

## Author Contributions

CY and SP proposed this study idea concept, designed the experiments, and modified the manuscript. XZ and LL were responsible for performing the experiments and writing the original manuscript. YX, FL, YH, and DH were responsible for finding relevant literature and materials. All authors contributed to the article and approved the submitted version.

## Conflict of Interest

The authors declare that the research was conducted in the absence of any commercial or financial relationships that could be construed as a potential conflict of interest.

## Publisher’s Note

All claims expressed in this article are solely those of the authors and do not necessarily represent those of their affiliated organizations, or those of the publisher, the editors and the reviewers. Any product that may be evaluated in this article, or claim that may be made by its manufacturer, is not guaranteed or endorsed by the publisher.

## Footnotes

1
http://www.ppmi-info.org
